# A label-free and low-power microelectronic impedance spectroscopy for characterization of exosomes

**DOI:** 10.1371/journal.pone.0270844

**Published:** 2022-07-08

**Authors:** Leilei Shi, Leyla Esfandiari

**Affiliations:** 1 School of Arts and Sciences, Carolina University, Winston-Salem, North Carolina, United States of America; 2 Department of Biomedical Engineering, College of Engineering and Applied Science, University of Cincinnati, Cincinnati, Ohio, United States of America; 3 Department of Electrical Engineering and Computer Science, College of Engineering and Applied Sciences, University of Cincinnati, Cincinnati, Ohio, United States of America; 4 Department of Environmental and Public Health Sciences, College of Medicine, University of Cincinnati, Cincinnati, Ohio, United States of America; University of Sharjah, UNITED ARAB EMIRATES

## Abstract

Electrical Impedance Spectroscopy (EIS) is a non-invasive and label-free technology that can characterize and discriminate cells based on their dielectric properties at a wide range of frequency. This characterization method has not been utilized for small extracellular vesicles (exosomes) with heterogenous and nano-scale size distribution. Here, we developed a novel label-free microelectronic impedance spectroscopy for non-invasive and rapid characterization of exosomes based on their unique dielectric properties. The device is comprised of an insulator-based dielectrophoretic (iDEP) module for exosomes isolation followed by an impedance spectroscopy utilizing the embedded micro-electrodes. This device is capable of distinguishing between exosomes harvested from different cellular origins as the result of their unique membrane and cytosolic compositions at a wide range of frequency. Therefore, it has the potential to be further evolved as a rapid tool for characterization of pathogenic exosomes in clinical settings.

## Introduction

Small extracellular vesicles (exosomes) are nano-scaled vesicles (40–150 nm) produced by many cell types and can circulate in almost all biofluids, including blood, urine, breast milk, cerebral fluids, and saliva.[1–8] Exosomes are shown to carry cell-specific cargos of proteins, lipids, and nucleic acids [9], and they could either fuse with the target cell through endocytosis or bind to the cell through receptor-ligand interaction in order to transfer the proteins and nucleic acids to the recipient cell [10]. Since exosomes biochemical composition and function can be mapped back to their parental cells [11], they have been considered as circulating biomarkers for early diagnosis of various diseases in liquid biopsy [11,12]. The conventional methods for characterization of exosomes, including transmission electron microscopy (TEM), nanoparticle tracking analysis (NTA), and western blots, are mainly based on exosomes morphology, size distribution, and immunoaffinity of their specific surface proteins. However, these techniques either fail to provide enough information about exosomes’ biochemical properties and/or are low throughput and time-consuming to operate.

Electrical impedance spectroscopy (EIS) is a label-free and non-invasive method for single cell characterization based on the cells’ unique dielectric properties at a wide range of frequency [13]. Generally, at low frequencies (< 1MHz), cells are insulating and resisting the current flowing into their interior and thus, the impedance is dominated by cell’s volume. At intermediate frequencies (1–10 MHz), cell’s membrane exhibits a capacitive response due to the polarization of the interface between the membrane and the surrounding medium. As the frequency further increases (> 10MHz), the electric field can penetrate into the cell’s membrane, and thus, the impedance signal reflects the cells’ cytoplasm properties [14–16]. The most common EIS technology for single cell measurement is microfluidic flow cytometry (MFC), in which an alternative current (AC) is applied by embedded microelectrodes to establish an electric field in the channel filled with conductive fluid [17,18]. As the cell passes through the channel, fluctuation of electric current is detected at different frequencies, and thus, providing information with regards to the cell’s impedance. Another common strategy is based on the static state impedance measurement in which a single cell is immobilized between a pair of sensing electrodes by either mechanical trapping, electrical trapping, or optical tweezers [19–23]. After positioning the cell at the center of sensing electrodes, its impedance will be measured by the embedded micro-electrodes at a wide range of frequency. Although these technologies can characterize a single cell, it will be challenging to directly adapt them for the characterization of a single nanoscale vesicle. One of the challenges is that the scale of the channel and electrodes must be miniaturized to nanoscale dimensions to achieve a reliable sensitivity, which puts restrictions on the device resolution and fabrication processes [19]. In addition, there are several other challenges remain including passing a single vesicle one at a time through a highly resistive channel utilizing a high pressure pump, and manipulating a single vesicle to the desired position in channel for precise sensing.

We have previously reported an insulator-based dielectrophoretic (iDEP) device to trap exosomes from biofluids by applying a low DC field across an array of micropipettes [24,25]. In addition, we have recently developed a proof-of-concept EIS system using the iDEP micropipette system with sensing electrodes manually positioned across the trapped particles by a micromanipulator [26], and a microchip comprised of microfabricated triangular posts on a substrate for entrapment of nanoscale particles using the DEP force and coplanar microelectrodes for impedance measurements of the particles [27]. The integrated microchip device could differentiate sub-micron particles including 100 nm polystyrene beads and liposomes as their impedance was measured using an AC field at 1 kHz to 10 MHz [27]. Here, the EIS microchip has been further improved by combining the micropipettes iDEP device with embedded micro-electrodes to differentiate and characterize exosomes from different cellular origins within 15 minutes. The device operates by initially trapping a cluster of exosomes (~ 1 million exosomes) at close proximity of the micropipette tips based on the force balance of three electrokinetic forces as we previously reported [24,25], followed by their characterization utilizing an on-chip EIS at a broad frequency spectrum (1kHz to 50 MHz). Compared to our previous EIS microchip, the applied electric field in this device is ten times lower for exosome trapping due to the smaller pore opening of the micropipettes, and thus, decreasing the possibility of denaturation of the vesicles. Also, the disposable and cost-effective embedded micropipettes, along with the open channel configuration could improve the robustness of sample processing step (i.e., for adding/removing reagents or removing bubbles) and omit the channel blockage issue. Furthermore, the reusable sensing module could significantly decrease the cost for each measurement. This integrated isolation and characterization platform demonstrated the capability to discriminate exosomes harvested from different cellular origins at the intermediate and high frequency range (10–50 MHz). The difference between the impedance of the exosomes could be correlated to their unique dielectric properties and can potentially be linked to their unique membrane and cytosolic compositions as they secreted from various parental cells. Thus, the device could be further evolved to a simple yet powerful tool for label-free characterization of nanovesicles in the clinical settings.

## Methods

### Materials

All chemicals were purchased from Sigma-Aldrich (St. Louis, MO, USA) unless otherwise noted. 100 nm carboxylic acid polystyrene (COOH-PS) beads were obtained from Phosphorex Inc. (Hopkinton, MA, USA). Fluorescently labeled 100 nm liposomes were purchased from FormuMax Scientific Inc. (Sunnyvale, CA, USA). Exosomes harvested from hTERT-immortalized mesenchymal stem cells, A549 non-small cell lung cancer (NSCLC) cell lines, HCT-116 Colorectal Carcinoma cell lines, and LNCap Clone Prostate Carcinoma cells were purchased from ATCC (Manassas, VA, USA). Gold etchant (Type TFA) and chromium etchant (1020AC) were obtained from Transene Company Inc. (Danvers, MA, USA). Photoresist AZ5214E, AZ4620, and developer AZ917 MIF, AZ400K were purchased from Integrated Micro Materials (Argyle, TX, USA). Polyimide (PI2610) and adhesion promoter MV652 were obtained from Hitachi DuPont MicroSystems LLC (Parlin, NJ, USA). 100 mm JGS2 fused silica glass wafers with 500 μm thickness were purchased from MES Supplies LLC (Tucson, AZ, USA). Platinum plated titanium electroplating anode and 24K non-cyanide gold electroplating solution were obtained from Gold Plating Services (Kaysville, UT, USA). The transparent glue was obtained from Elmer’s Products (Westerville, OH, USA).

### Preparation of solution containing sub-micron particles

Electrolyte solutions containing different potassium chloride (KCl) concentrations (1, 10, 100, and 500 mM) and 1×PBS were prepared at pH 7.0. The conductivities of electrolyte solutions were measured utilizing a conductivity meter (Oakton Cond 6+) as: 0.11 S/m for 1 mM KCl, 0.30 S/m for 10 mM KCl, 1.39 S/m for 100 mM KCl, 5.88 S/m for 500 mM KCl, and 1.62 S/m for 1×PBS.

100 nm COOH-PS beads were re-suspended into 1×PBS to the final concentration of 2.3×10^12^ /mL. 100 nm liposomes were re-suspended into 1×PBS to the final concentration of 1.9×10^11^ /mL. Exosomes secreted from different cellular origins, including hTERT Mesenchymal Stem cells, A549 Non-small Lung Carcinoma cells, HCT-116 Colorectal Carcinoma cells, and LNCap Clone Prostate Carcinoma cells, were distributed in 1×phosphate-buffered saline (PBS) at the final concentration of 1×10^9^ /mL.

### Device layout and fabrication

**Fig 1:**
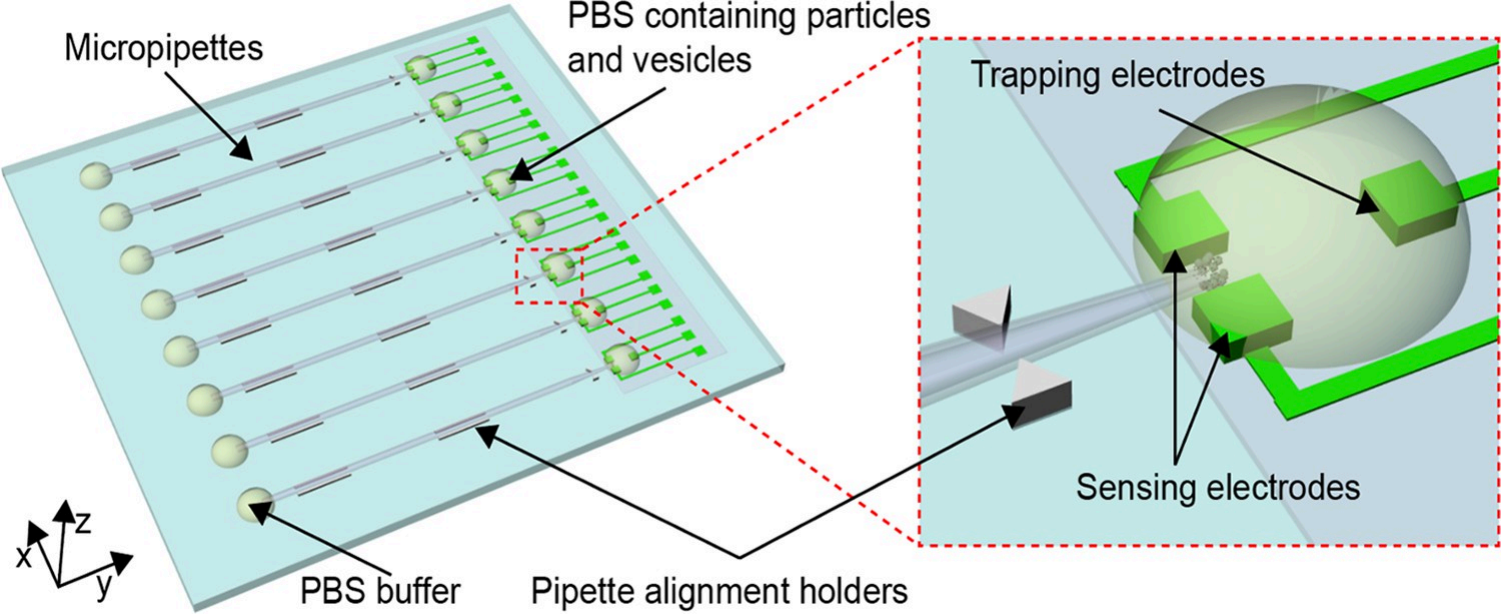
Schematic of the microchip. The microchip consists of three modules: glass iDEP micropipettes for particle trapping, the embedded micro-electrodes for impedance measurement, and PMMA substrate with holders for pipettes alignment.

The LOC device was designed with AutoCAD 2018. A schematic illustration of the microchip is presented in Fig 1. The device contains three modules: glass micropipettes for particle trapping, the embedded micro-electrodes for impedance measurement, and PMMA substrate with alignment holders for placing the micropipettes’ tips in an exact position with respect to the micro-electrodes.

Micropipettes with ~2 μm pore diameters were fabricated with the laser-assisted puller, Sutter-2000. A thick wall borosilicate glass capillary (BF-100-50-15) was positioned at the puller, and a laser beam was focused into the center of the capillary to melt the glass. At the end of the program, two identical pores were formed as the glass capillary separated at the center. The puller program with the following settings was used: Heat 350, Filament 4, Velocity 18, Delay 200, Pulling 0. The diameters of pipettes were approximated by comparing their ionic conductance with the ionic conductance of the same size pipettes purchased from World Precision Instruments, Inc and evaluated microscopically.

**Fig 2:**
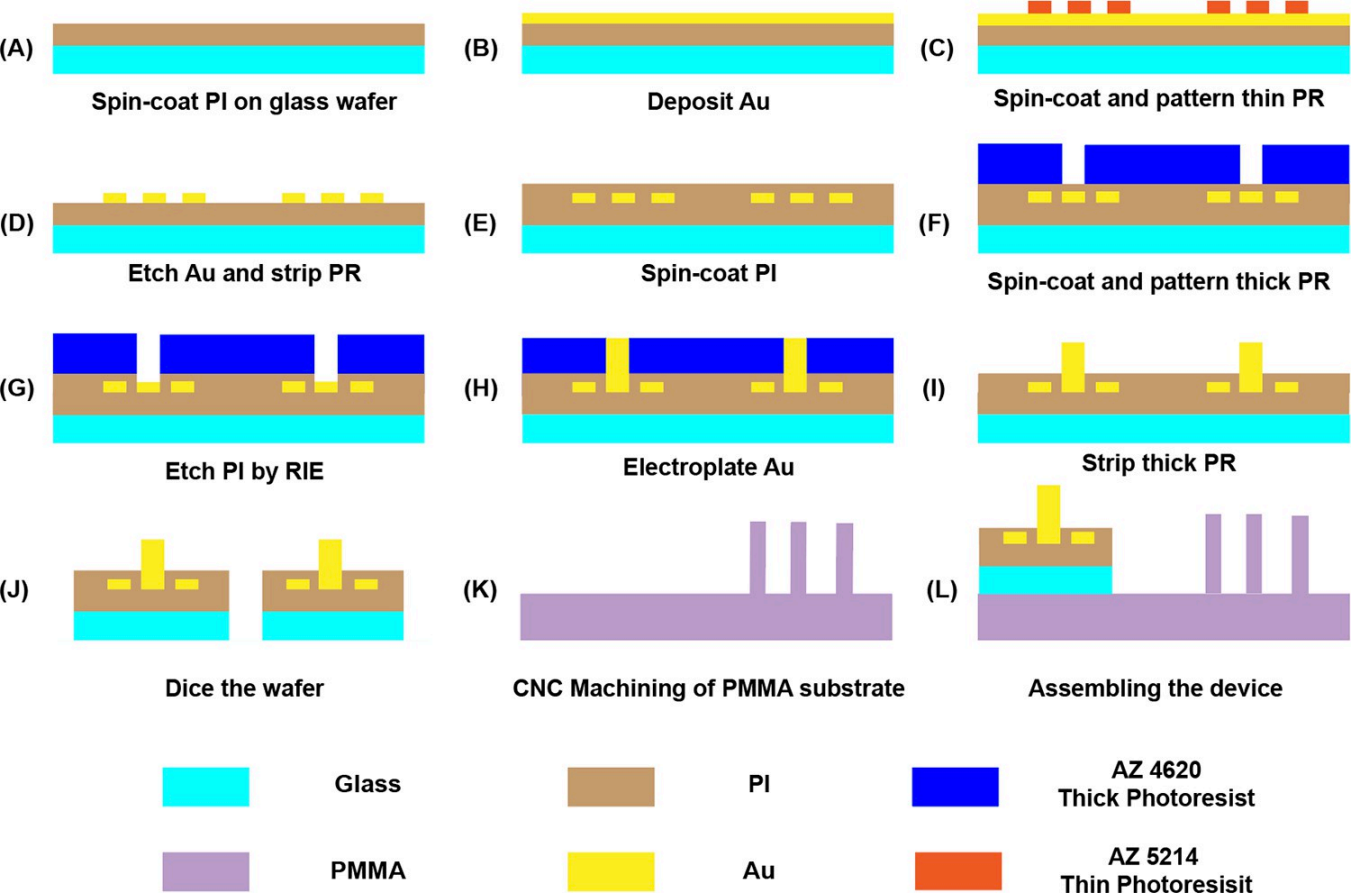
Step by step fabrication procedure of proposed LOC device.

The embedded micro electrodes were fabricated using the photolithography technique. Firstly, a layer of PI2610 was coated on a glass wafer to increase the adhesion of gold to the substrate. Prior to the PI2610 spin-coating, an aminosilane-based adhesion promoter VM652 was applied and spin-coated at 2000 rpm for 30 seconds to enhance the adhesion of PI2610 to the glass wafer. PI2610 was then spun coated at 5000 rpm for 30 seconds and baked at 350°C for 40 minutes to obtain a layer of PI with 1 μm thickness (Fig 2A). Afterwards, metal (10 nm Cr and 100 nm Au) was deposited on the PI-coated substrate using the E-beam evaporator (Fig 2B). The deposited metal was patterned by photolithography with AZ5214E as positive photoresist and MIF 917 as developer (Fig 2C). The redundant metal was etched by gold etchant and chromium etchant (Fig 2D). Afterwards, another layer of PI2610 was coated on top of the electrodes to protect the connecting wires (Fig 2E). A layer of thick photoresist AZ4620 with 27 μm thickness was coated on the device using double spin-coating method. Specifically, AZ4620 was initially spin-coated at 2500 rpm for 60s, followed by a baking process at 110°C for 90s. Afterwards AZ4620 was spin-coated at 1600 rpm for 60s followed by a 110°C baking for 170s. Next, the AZ4620 photoresist layer was patterned to create windows for sensing electrodes (40×40 μm), trapping electrodes (400×400 μm), and soldering pads (2×3 mm) (Fig 2F). PI2610 covered on those areas was removed by an oxygen reactive ion etching (O_2_-RIE) process with applied 150 W power for 15 minutes (Fig 2G). An electroplating process was used to obtain gold electrodes with 20 μm height (Fig 2H). The wafer was immersed into the gold electroplating solution with an applied 1.5 V DC voltage for 19 hours at 45°C. Afterwards, the covered photoresist on the wafer was removed by acetone (Fig 2I), and the wafer was diced to individual sensing chip by MPE Inc. (Greenville, TX, USA) (Fig 2J).

The PMMA substrate with alignment holders was fabricated by computer numerical control (CNC) micro-machining technique. The substrate was designed using the AutoCAD 2018 software to create a computer-aided design (CAD) file. The MasterCAM software (CNC software, CT, USA) was then employed as computer-aided manufacturing (CAM) program to convert the CAD files into numerical control (NC) programming language for running the CNC micro-milling machine. Finally, the PMMA substrate was micromachined by a 5100-S CNC milling machine (Microlution, IL, USA) (Fig 2K). The diced individual sensing chip with patterned electrodes was manually aligned and bonded with the PMMA substrate using a transparent glue under the microscope (Fig 2L).

### Pipette loading and impedance measurement

**Fig 3:**
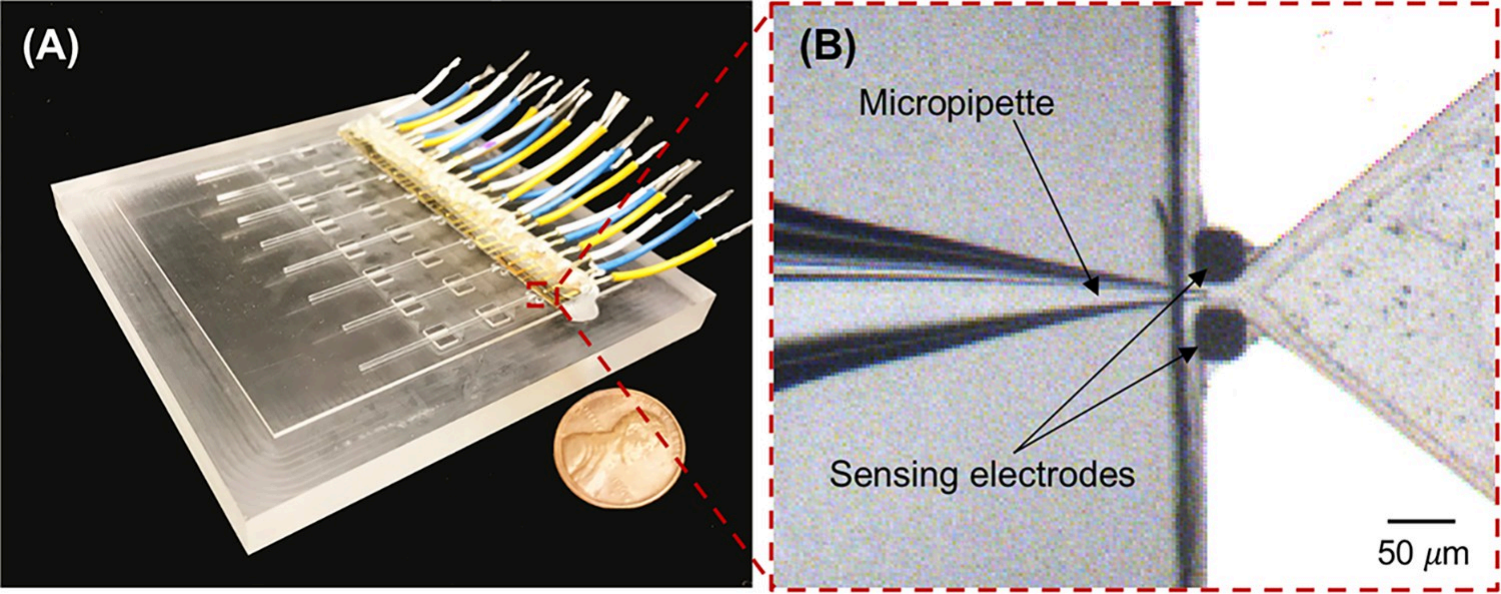
The picture and microscopic image of the microchip. (A) The integrated microelectronic device. (B) Bright-field microscopic image of the alinged micropipette tip in between two sensing electrodes.

Micropipettes were backfilled with 1×PBS buffer via a 33-gauge Hamilton syringe needle, and were placed on the PMMA substrate to align the position of the micropipettes’ tips in the middle of the embedded sensing micro-electrodes (Fig 3).

10 μL of electrolyte solution containing different particles including 2.3×10^12^ /mL COOH-PS beads, 1.9×10^11^ /mL liposomes, and 1×10^9^ /mL exosomes were injected into the tip side chamber of the micropipettes. Meanwhile, same amount of 1×PBS buffer was added to the chamber facing the base of pipettes. 5V/cm DC bias was applied across the trapping electrodes using a Keithley 2220G-30-1 voltage generator for 10 minutes to accumulate particles at the tip of the pipettes. The microscopic images were recorded using an inverted microscope, Nikon Eclipse TE2000-S, equipped with a high-resolution camera, Andor NeoZyla 5.5, at a capturing frequency of 100 frames per second.

**Fig 4:**
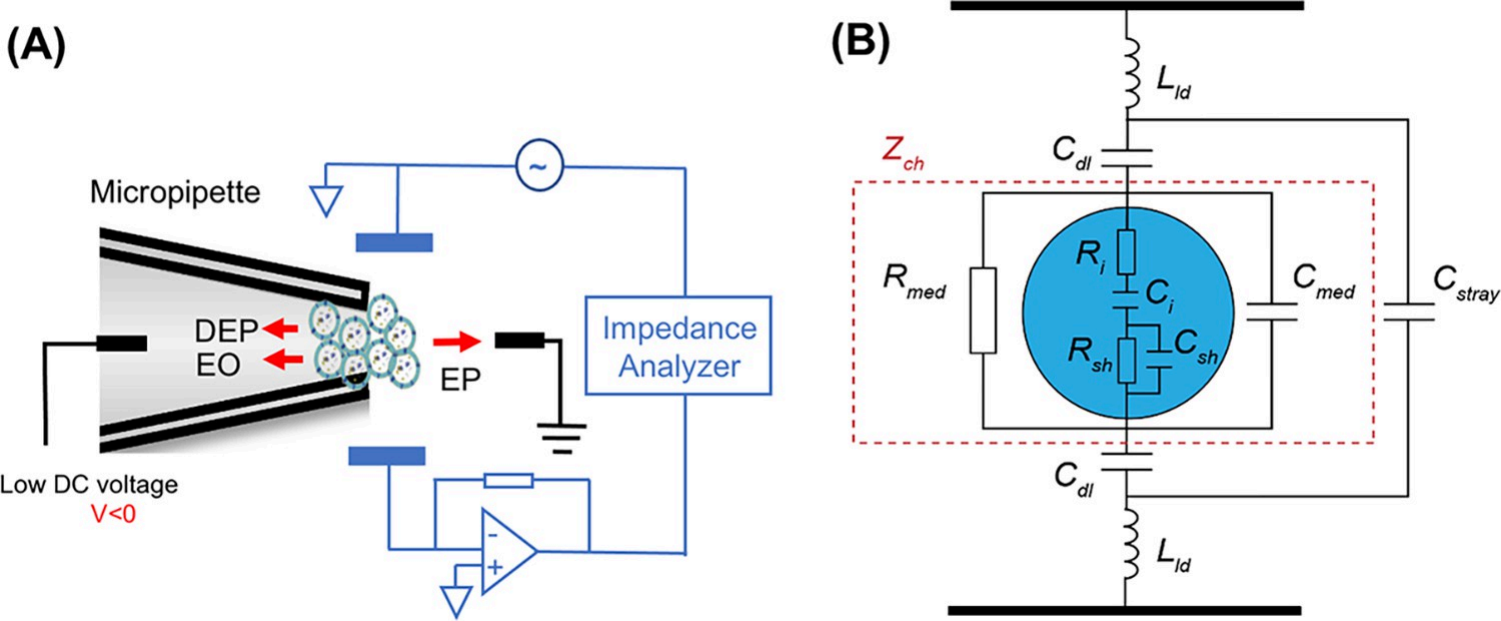
The schematic illustration and the equivalent circuit of the impedance measurement system. (A) The schematic of the impedance measurement system. Exosomes were trapped at the tip of the micropipette due to the balance of dielectrophoresis (DEP), electrophoresis (EP), and electroosmosis (EO) forces. The impedance of the vesicles was characterized by an impedance analyzer at a broad frequency spectrum (1kHz to 50 MHz) utilizing the sensing electrodes. (B) Constructed model of an equivalent circuit of the system where *L*_*ld*_, *C*_*stray*_, *C*_*dl*_, *Z*_*ch*_, *R*_*i*_, *C*_*i*_, *R*_*sh*_, *C*_*sh*_, *R*_*med*_, and *C*_*med*_ represent the lead inductance, stray capacitance, electrical double-layer capacitance, channel impedance, inner core resistance, inner core capacitance, shell resistance, shell capacitance, suspending medium resistance, and suspending medium capacitance respectively.

Impedance measurement of trapped particles was conducted utilizing the digital impedance analyzer (HF2LI, Zurich Instrument) as an AC field with a peak amplitude of 100 mV swept from 1 kHz to 50 MHz to record the magnitude and phase components at each frequency (Fig 4A). The broad frequency spectrum (1KHz -50 MHz) was chosen to cover nanovesicles’ intrinsic properties including their membrane capacitance and luminal conductance while avoiding the parasitic capacitance at ultrahigh frequency (> 50 MHz) [14–16]. The impedance signals were recorded at a sampling rate of 225 samples/sec and each measurement was repeated at least three times. The data analysis was obtained as previously reported by our group [26,27]. The impedance was normalized based on the ‘opacity’ concept to rule out the effect of the particles’ number as shown in Eq (1) [28–33].

$$O_{f}=\frac{Z(f)}{Z(0.5\;MHz)}$$ (1)

where *Z*(*f*) and *Z*(0.5 *MHz*) are the impedance measured at frequencies higher than 0.5 MHz and at 0.5 MHz respectively.

Statistical analysis was performed using the student’s t-test and two-way analysis of variance. The difference with p-values less than 0.05 were considered as significant. After each impedance measurement, the micropipettes were disposed, and the substrates with embedded microelectrodes were cleaned based on the established Lab-on-Chip device cleaning protocol to be reused with freshly made micropipettes [34].

### Theoretical modeling and equivalent circuit

An equivalent circuit model (Fig 4B) was constructed to demonstrate the physical principle of the impedance measurement system [15,35]. In this model, the channel impedance *Z*_*ch*_ is in series with an electrical double layer capacitance *C*_*dl*_ and is in parallel with a stray capacitance *C*_*stray*_ [20,36,37]. In addition, a lead inductance (*L*_*ld*_*)*, which is introduced by the impedance analyzer connecting cables, is included in the equivalent circuit. When single-shell vesicles, such as liposomes and exosomes, are entrapped in between a pair of sensing electrodes, the channel impedance *Z*_*ch*_ could be represented with an equivalent circuit, in which the suspending medium is connected in parallel with the vesicles [35]. The vesicles are modeled as a resistor *R*_*i*_ and a capacitor *C*_*i*_ in series (inner core) in combination with a resistor *R*_*sh*_ and a capacitor *C*_*sh*_ in parallel (shell).

The values of *C*_*dl*_, *C*_*stray*_, and *L*_*ld*_ were obtained via measurements of electrolyte solutions with known electrical properties, followed by fitting into the combination of constant phase element and Cole-Cole model [38,39]. Fitting parameters that were used throughout this theoretical modeling were *C*_*dl*_ = 80 pF, *C*_*stray*_ = 7 pF, and *L*_*ld*_ = 6 μH, respectively.

Channel impedance *Z*_*ch*_ for electrolyte solutions was calculated based on Maxwell’s Mixture Theory (Eq 2) [35,40].

$${\tilde{Z}}_{ch}=\frac{1}{j\omega {\tilde{\varepsilon }}_{med}G_{f}}$$ (2)

where ${\tilde{\varepsilon }}_{med}$ is the complex permittivity of the medium, *ω* is the angular frequency, and *G*_*f*_ is the geometrical constant of the system.

The geometrical constant *G*_*f*_ in our system could be approximately calculated as $G_{f}=\frac{A}{g}$ [35], where *A* is the electrodes surface area and *g* is the gap distance between a pair of electrodes. Since in our model the electrode area is *A* = 800 μm^2^, and gap distance is *g* = 40 μm, *G*_*f*_ was calculated as 20 μm.

The complex permittivity of the medium is given by:

$${\tilde{\varepsilon }}_{med}=\varepsilon_{m}-j\frac{\sigma_{m}}{\omega }$$ (3)

where *ε*_*m*_ and *σ*_*m*_ are permittivity and conductivity of electrolyte solutions, respectively.

## Results and discussion

### Impedance measurement of solution with various ionic strengths

**Fig 5:**
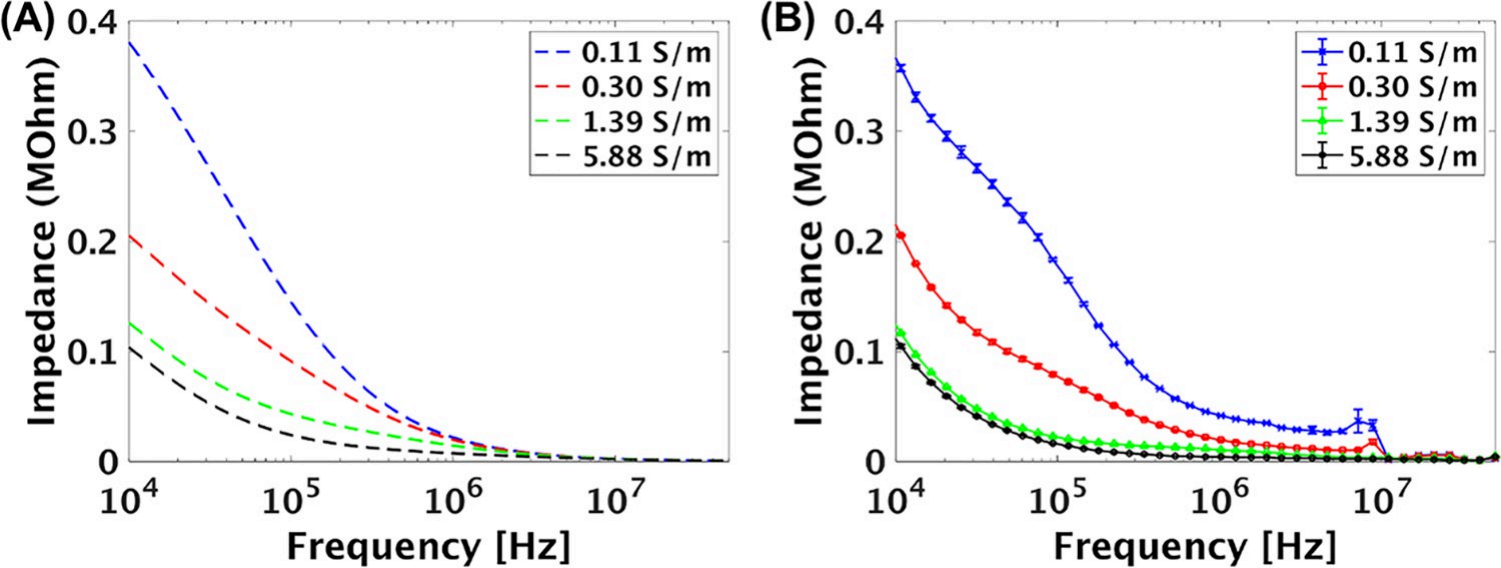
The modeling and experimental results showing the impedance of soluitons with different conductivities as a function of frequency. (A) The results obtained from an equivalent circuit model showing the impedance of solutions with different conductivities as a function of frequency. (B) The corresponding experimental results showing the impedance of solution with different conductivities as a function of frequency.

The impedance of the solutions with different ionic strength were modeled using the equivalent circuit as a resistor and a capacitor connected in parallel and the results were plotted in Fig 5A. The theoretical results illustrate that the absolute value of impedance decreased as the frequency increased for all solutions. As the frequency increased from 10 kHz to 50 MHz, the capacitive reactance decreased, and thus, the overall impedance decreased for all the electrolyte solutions which was consistent with the previously reported analysis [21,27,41,42]. The theoretical results were followed by experimental observations (Fig 5B) in which similar trends for each ionic strength was obtained. Hence, the established equivalent circuit model is reliable for predicting the impedance of the system. In addition, a sharp decrease of impedance was observed in the empirical results at around 10 MHz. This could be attributed to the so-called Debye-Falkenhagen effect that caused the conductivity of the electrolyte solutions to increase rapidly at around 10 MHz [43,44].

### Impedance measurements of sub-micron particles

**Fig 6:**
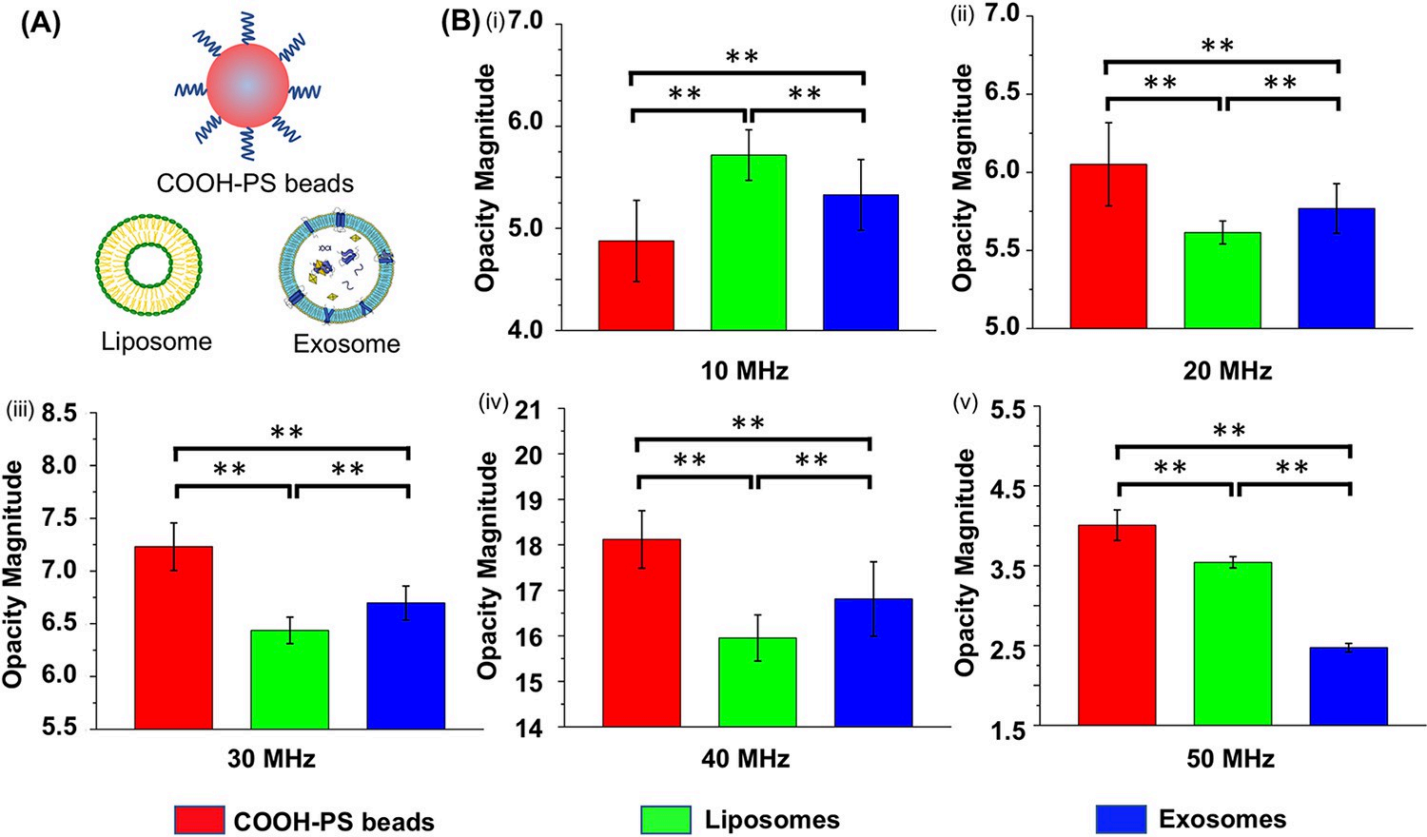
The opacity magnitude comparison of different sub-micron particles. (A) Schematic illustration of different sub-micron particles, including COOH-PS beads, liposomes, and exosomes harvested from hTERT mesenchymal stem cells. (B) The opacity magnitude comparison among COOH-PS beads, liposomes, and exosomes from 10MHz to 50 MHz. The error bars represented standard deviation. p values p<0.05(**) considered as a significant difference.

To study the capability of the device to differentiate sub-micron particles based on their unique dielectric properties, 10 μL of 1xPBS buffer containing COOH-PS beads, liposomes, and exosomes harvested from hTERT mesenchymal stem cells (Fig 6A) were injected in the tip side of three micropipettes, followed by entrapment and impedance measurements at a wide frequency (1 kHz to 50 MHz). The impedance of particles was normalized based on opacity magnitude [28–33], and plotted in Fig 6B. The results illustrate that exosomes harvested from hTERT stem cells, liposomes, and COOH-PS beads with similar size distribution (~100 nm) could be distinguished at 10–50 MHz. When comparing their opacity magnitudes, we observed that the opacity of COOH-PS beads was lower than liposomes and exosomes at 10 MHz which was in line with our previous report [27]. We suggested that COOH-PS beads have a higher surface capacitance due to the modification of negatively surface charged carboxylic acid groups, and hence, resulting in a lower impedance signal at 10 MHz. However, as the frequency increased to 20 MHz and above, the opacity of COOH-PS beads became higher than liposomes and exosomes. This shift in opacity trend could be attributed to the fact that the surface capacitance became insignificant as the frequency increased to 20MHz and above, and thus, the bulk polystyrene material with high resistance showed a high impedance signal. In addition, when comparing liposomes and exosomes, we observed that the opacity of exosomes was lower than liposomes at 10 MHz. We postulated that the presence of embedded proteins on the membrane of exosomes increased the membrane capacitance, and thus, resulting in a lower impedance signal [35]. However, as the frequency increased to the range of 20–40 MHz, we observed that the opacity of exosomes was higher than liposomes. We hypothesized that at frequencies higher than 10 MHz, the electric field could penetrate through the membrane of the nanovesicles, and thus, the system mainly measured their cytosolic impedance [14–16]. Since the protein and nucleic acids encapsulated in the lumen of exosomes have relatively lower permittivity and higher conductivity than the electrolyte solution in the lumen of liposome, the presence of protein and nucleic acids in exosomes increased the capacitive reactance and decrease the resistive reactance. Therefore, we suggested that the higher opacity of exosomes was attributed to the fact that the capacitive part was more dominant in their cytosolic impedance at the frequency range of 20–40 MHz. Interestingly, as the frequency increased to 50 MHz, we observed a shift in opacity trend between exosomes and liposomes, and the opacity of exosomes was lower than liposomes. We postulated that the capacitive characteristic of exosomes’ lumen became insignificant as the frequency increased to 50 MHz, and thus, the resistive part became more dominant.

### Impedance measurements of exosomes harvested from different cellular origins

**Fig 7:**
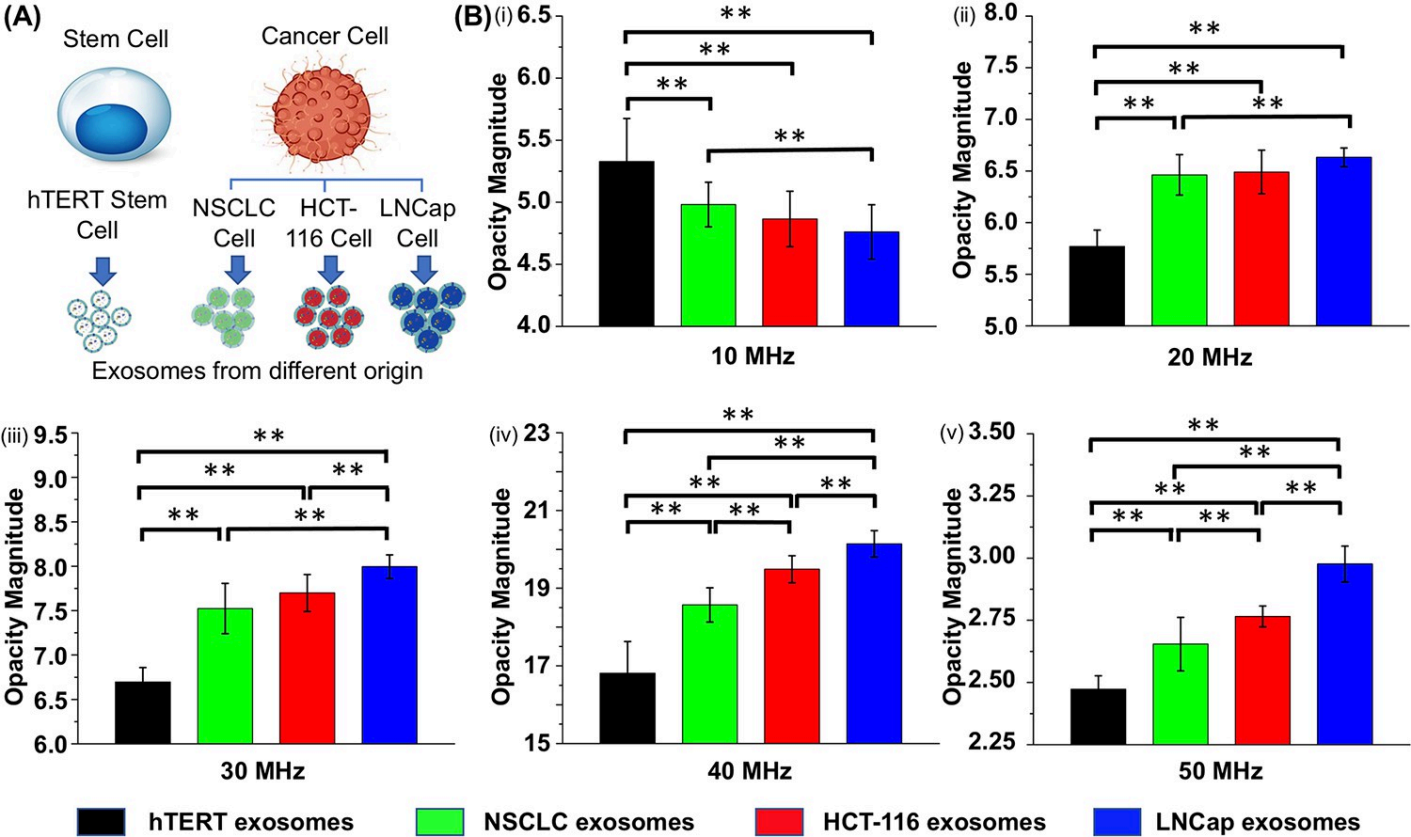
The opacity magnitude comparison of exosomes harvested from different cellular origins. (A) Schematic illustration of exosomes harvested from hTERT mesenchymal stem cells and three different carcinoma cell lines (HCT-116, LNCap, and NSCLC obtained from ATCC Inc.). (B) The opacity magnitude comparison of exosomes harvested from different cell lines, including hTERT, HCT-116, LNCap, and SNCLC. The error bars represent the standard deviation, and p<0.05(**) considered as a significant difference.

Since exosomes are circulated in almost all biofluids and they could provide crucial molecular information about their parental cells, they have the potential to be utilized as biomarkers for disease diagnosis in liquid biopsy [45,46]. Thus, discrimination of exosomes harvested from different cellular origins has gained particular interest. Although the current biochemical characterization methods such as Western blot and RNA sequencing are highly accurate for discrimination of exosomes, they need lysis or labeling steps prior to characterization, which not only increase the processing time and cost, but also break the structure of the vesicles. Here, we demonstrated the capability of the EIS system to rapidly and non-invasively differentiate exosomes harvested from different cellular origins based on their unique dielectric properties. In our experiments, four different commercially available exosomes were selected to ensure the purity of the sample for device performance validation. 10 μL of 1xPBS buffer containing exosomes harvested from hTERT Mesenchymal Stem cells, A549 Lung Carcinoma cells, HCT-116 Colorectal Carcinoma cells, and LNCap Clone Prostate Carcinoma cells (Fig 7A), was injected into the chamber facing the four pipette’s tip. A 5 V/cm DC bias was applied across the micropipettes for 10 minutes to entrap exosomes at the tips, followed by the impedance measurement of the entrapped exosomes and plotted histogram of their magnitude opacities as shown in Fig 7B. The analysis demonstrated that exosomes secreted from hTERT stem cells could be distinguished from the exosomes harvested from the other three carcinoma cells at frequency range of 10–50 MHz; however, exosomes harvested from the other three carcinoma cells could be distinguished from each other at higher frequency range >40 MHz. Specifically, exosomes secreted from LNCap cells and NSCLC cells could be discriminated at the frequency range of 10–50 MHz; exosomes secreted from HCT-116 cells and LNCap cells could be distinguished at the frequencies above 30 MHz; and exosomes secreted from HCT-116 cells and NSCLC cells could be differentiated at frequencies above 40 MHz. The other interesting observation was that the opacity increased from 10–40 MHz and decreased from 40–50 MHz for all types of exosomes. To investigate the origin of the opacity variation at different frequencies, pure PBS solution was tested which showed the same opacity trend (data not shown). It is concluded that the fluctuation of the opacity value with respect to frequency is mainly caused by the intrinsic impedance of the measurement system and thus, it is important to analyze the relative opacity of each sample with respect to each other at every frequency. We also postulate that the differentiable impedance signals between exosomes harvested from different origins were correlated to the difference between exosomes’ dielectric properties and could resemble their differences in membrane and cytosolic compositions. Specifically, the membrane impedance may be greatly affected by the variation of the phospholipids, embedded proteins, and their relative ratio. On the other hand, the cytosolic impedance could be affected by the difference between their encapsulated proteins and nucleic acids content. For the exosomes that could only be distinguished at frequency above 30 MHz or 40 MHz, we hypothesized that their dielectric properties have more similarities especially in terms of their membrane compositions. However, to precisely correlate the membrane and cytosolic composition of exosomes to their frequency-dependent impedance, more comprehensive and precise studies on exosomes’ molecular profiles, including genomic, proteomic, and lipidomic analysis need to be conducted, which will be the subject of our future study.

## Conclusion

A novel impedance microelectronic has been developed for rapid and label-free characterization of exosomes based on their unique dielectric properties. The device is capable of initially trapping a cluster of exosomes (~ 1 million exosomes) at the close proximity of the micropipettes’ tips based on the force balance of three electrokinetic forces; Followed by the characterization of the entrapped exosomes, as their impedance was measured by embedded micro-electrodes at a wide range of frequency (1KHz-50MHz) in situ. An equivalent circuit was built to model the impedance of electrolyte solutions with known dielectric properties. The theoretical model was validated by empirical observations, and the closely matched trends in the impedance results indicated that the established equivalent circuit was reliable for predicting the impedance of the system. In addition, we demonstrated the capability of the device to differentiate between different sub-micron particles, including liposomes, polystyrene beads, and exosomes with similar size, which provide an insight into the relationship between the particles’ dielectric property and their frequency-dependent impedance. Moreover, the device could distinguish between exosomes secreted from different cellular origins based on their unique dielectric properties. The results indicated a difference in their opacity at frequency 10 MHz and above, which most likely reflect on their unique compositions in terms of their membrane and cytosolic charge dependent contents. Furthermore, we observed that the opacity of exosomes secreted from HCT-116 cells have relatively smaller differences from both the exosomes secreted from LNCap cells and NSCLC cells. We postulated that the small differences in opacity reflect on their closely related dielectric properties, which could be potentially linked to their relatively similar membrane compositions. However, further studies on exosomes’ molecular profiles will be the subject of our future work to obtain a more precise correlation between the composition of exosomes and their frequency-dependent impedance and hence, their unique dielectric properties.
